# Participatory variety selection of groundnut (*Arachis hypogaea* L.) in Taricha Zuriya district of Dawuro Zone, southern Ethiopia

**DOI:** 10.1016/j.heliyon.2022.e09011

**Published:** 2022-02-24

**Authors:** Demelash Bassa Belayneh, Yasin Goa Chondie

**Affiliations:** Crop Work Process, Areka Agricultural Research Center, SARI, P. O. Box 79, Areka, Ethiopia

**Keywords:** Baby trial, Groundnut, Mother trial, Participatory, Selection, Variety

## Abstract

Groundnut is a leguminous seed that contains a lot of oil and protein with high energy content. However, improved varieties were hardly evaluated based on farmers' preference criteria and their participation. Therefore, a participatory variety selection was carried out in Taricha Zuriya district in Dawuro Zone on the mother-baby approach on farmers' fields in the 2019 and 2020 main cropping seasons, aiming at evaluating the performance of groundnut varieties through farmers' participation and assessing their preference criteria. Six released groundnut varieties were tested using a randomized complete block design with four replicates at farmers' fields. The mother trial was done at one model farmer field (all four replications on one field), whereas the baby trials were done at four farmers' fields by considering farmers as replication per village. Combined analysis of variance for two years showed a highly significant (P < 0.001) to significant (P < 0.01) differences among groundnut varieties for grain yield, days to 50 % flowering, days to maturity, seeds per pod, pods per plant, 100 seed weight, except for both stand count at emergence and harvest. Among the tested varieties, BaHajidu (1805.84 kg ha^−1^) was identified as the best yielding groundnut variety, followed by Bulki-01 (1805.50 kg ha^−1^) and Werer-963 (1780.0 kg ha^−1^), respectively, while Werer-962 variety has a lower yield (1536.30 kg ha^−1^). Bulki-01 (96), BaHajidu (90), and Werer- 963 (76) obtained higher score values as preferred by farmers, whereas lower score values were observed for Manipinter variety (45). The aforementioned varieties Bulki-01, BaHajidu and Werer-963 were also preferred using selection criteria set by farmers as 1^st^, 2^nd,^ and 3^rd^ in rank order respectively. Hence, based on farmers’ preference values and biological data, these three groundnut varieties were recommended for pre-extension demonstration and large-scale production in Dawuro Zone and areas with similar agro-ecologies.

## 1 Introduction

Groundnut (*Arachis hypogaea* L., 2n = 4x = 40, AABB) is a self-pollinating allotetraploid legume crop belonging to the Fabaceae family (Janila et al., 2013). It is considered to be the most important monoecious annual legume used as human food, forage, and source of income in Sub-Saharan Africa (Alemayehu et al., 2014; Ajeigbe et al., 2015). It is an oilseed and grain crop that ranked 4th oilseed crop and 14th food crop in the world (Ahmed et al., 2016). The botanical name for groundnut, *Arachis hypogaea,* is derived from two Greek words, *Arachis* meaning legume and hypogaea meaning underground, referring to the formation of pods in the soil (ICAR, 2002). Globally, groundnuts are grown on 27.66 million hectares, with a total annual production of 43.98 million tons (FAOSTAT, 2018). It is mainly an annual self-pollinating legume and the main groundnut-producing countries in the world are India (20.97%), China (16.35%), Nigeria (9.68%), and Sudan (8.37%) (FAOSTAT, 2018). Groundnuts are commonly produced in Ethiopia for food, cash income, and as animal feed. It is solely grown by small-scale holder farmers in the lowland and drought-prone areas of the country. The estimated annual groundnut production in Ethiopia was about 145,191.45 tons from 80,841.57 ha of production area (CSA, 2018). Currently, groundnuts are widely produced in the Oromia Region, accounting for 59.2% of the total national production, followed by Benishangul-Gumuz (24.83%), Amhara (7.43%), Harari (3.29%), and the Southern Nation and Nationalities People (1.29%) regions (CSA, 2018). Groundnut seed is rich in 40–50% of fat, 20–50% of protein, 10–20% of carbohydrate, vitamins, and minerals; and provides 567 calories per 100 g (Ahmed et al., 2016).

This high-energy value, protein, and minerals make it an abundant source of nutrients at a low cost. About two-thirds of the world's groundnut seeds serve for the production of oil which is used for cooking, salad oils, and margarine, and lower quality oils are used in soap manufacture (Pradhan, 2011). Groundnut is one of the five oil crops widely cultivated in Ethiopia (Kudama, 2013) by the traditional farming communities in rain-fed conditions.

Besides its/nutritional value, it also has environmental benefits for farmers. Groundnut improves soil fertility by fixing nitrogen and thus increases the yield of other crops when used in rotation or intercropping (Ajeigbe et al., 2015). Growing demand in both domestic and export markets could also provide a source of cash for smallholder producers. The adoption of this crop is less as compared to the nutritive value and maintenance of soil fertility (Ahmed et al., 2016). Currently, the crop is becoming one of the high-value crops that are growing in the lowland areas of the Dawuro Zone in the south region, Ethiopia specifically in Taricha Zuriya areas.

Despite this, the yield of groundnut is still low in the country at 1.79 t ha^−1^ (FAOSTAT, 2018) and the low levels of adoption of productivity-enhancing technologies is one of the reasons for this yield level. The most common groundnut production constraint in Ethiopia in general and the southern region, in particular, were the lack of access to improved seeds, biotic, abiotic stress, and the use of low-yielding local varieties (Seltene et al., 2019). Hence, this study was developed to evaluate the performance of groundnut varieties through farmers' participation and to assess farmers’ selection criteria for the varieties.

## 2 Materials and methods

### 2.1 Description of the study area

The study was performed in the Wara hore farmers association of Taricha Zuriya district in the Dawuro Zone, which is located approximately 500 km southwest of Addis Ababa, the capital of Ethiopia, and 319 km from Hawassa, the capital of the Sidama region. It lies between 5°34′16.31″ N to 7°20′57.61″ N of latitude and 36°22′13.04″ E to 37°51′25.91″ E of longitude. The area is classified as 8.08% highland (>2400 m a.s.l.), 10.79% midland (1800–2400 m a.s.l.) and lowland 81.13% (<1800 m a.s.l.) agro-ecological zones. The altitudes ranged from 500-3600 m a.s.l. The major soil type which comprises about 91.1 % in the zone is Leptosols.

### 2.2 Design, treatments, and procedures

This study evaluated six improved groundnut varieties (Sedi, BaHajidu, Manipinter, Bulki-01, Werer-962, and Werer-963), sourced from Haramaya University (Table 1). Six groundnut varieties were laid out using a randomized complete block design within four replications for two successive years in 2019 and 2020. A mother trial (all four replications) was planted on one model farmer field and one replication each was planted on the other four farmers' fields (field 1: 7^o^ 10′N, 37^o^ 3′E; field 2: 7^o^ 11′N, 37^o^ 4′E; field 3: 7^o^ 13′N, 37^o^ 6′E and field 4: 7^o^ 14′N, 37^o^ 7′E with an altitude of 1500–1523 m a.s.l), which was considered as baby trials. Mother trial was planted to exploit researcher data whereas the baby trials were planted in farmers' fields to take farmers' preferences into account during variety evaluation. The plot size was 14.4 m^2^ of six rows of 4 m long in the spacing of 0.6 m and 0.1 m inter and intra rows, respectively. The harvestable area was four rows of 4 m long i.e. 9.6 m^2^. Four farmers' fields were used for evaluation and each farmer was used as replication. Sowing was done during the onset of rainfall. All the recommended cultural practices such as weeding, earthing up, fertilization, etc. were applied during the study period. The common fertilizer rate recommendation of legume crops i.e. half of 121 kg ha^−1^ NPS was used. NPS consists, Nitrogen, phosphorous, and sulfur in one pack (19 N–38 P2O5 +7S)

**Table 1:** Description of the groundnut varieties.

Variety	Year of release	MD	Seed color	Yield (t ha^−1^)	Maintainer/Breeder
Sedi	1993	137	Red	1.92	Werer ARC
BaHajidu	2012	126	Red	2.02	HU
Manipinter	1969 (Uganda)	155	Variegated	2.4	Werer ARC (Introduced)
Bulki-01,	2002	135	Light red	2.2	Werer ARC
Werer 962	2004	128	Red	2.1	Werer ARC
Werer 963	2004	128	Red	2.2	Werer ARC

MD = maturity days, HU = Haramaya University, ARC = Agricultural Research Center.

### 2.3 Data collection

Agronomic data were collected on a plot and plant basis from the mother trial. Data were collected on a plot basis and plant basis from the central four rows for all traits. Seeds per pod, pods per plant was evaluated on five plants randomly selected from the middle four rows of each plot while days to 50% emergence (DE 50%), days to 50 % flowering (DF 50%), days to 90% maturity (DM 90%), 100 seed weight (g) and grain yield were collected on a plot basis. The data recording for each trait (8) was carried out as follows.

1. Stand count at emergence: It was recorded as the number of plants in the plot at emergence
2. Stand count at harvest: It was recorded as the number of plants in the plot at harvestable maturity
3. Days to 50% flowering: It was recorded as the number of days from sowing to 50% of the plants in the plot started flowering
4. Days to maturity: It was recorded as the number of days from sowing to the stage when 90% of the plants in a plot have changed the color of their pods to yellow.
5. Number of pods per plant: This was determined as the mean value of five randomly sampled plants obtained by counting the total number of pods per plant.
6. Number of seeds per pod: The mean number of seeds per pod was obtained by counting the number of seeds collected from five mature pods from each five sampled plants.
7. Dry pod yield (kg ha^−1^): This was measured after harvesting the whole pods from the middle four rows of each plot and converted to kilograms per hectare after sun drying.
8. 100 seed weight: It was recorded by counting 100 seeds from a bulk of shelled seeds and weighed using a sensitive balance.

Diseases (leaf spot, root rot diseases, rust) and insect pests (termite) are the problems of groundnut at field conditions, and weevil is the storage pest reported in the region but we did not collect diseases and insect pests data in these trials. Farmers’ evaluation and selection data were collected on a plot basis from the four baby trials of each field. Farmers' field visits were organized at different stages of plant growth (at flowering, maturity) and harvest for farmers to share experiences and evaluate varieties in the trials. Two (2) Agricultural Development Agents, sixteen (16) men, and four (4) women farmers have participated in the selection process. The ranking procedure was explained to participants and then each criterion was ranked from 1 to 5 (5 = excellent, 4 = very good, 3 = good, 2 = fair and 1 = very poor) for each variety, the ranking was done on consensus where differences have been resolved through discussion. During the direct matrix ranking, farmers gave the importance (a relative weight) score of the selection criteria were ranked from 1 to 3 (3 = very important, 2 = important and 1 = less important) and a variety performance score for each trait of interest was given based on their level of importance based on the common agreement of evaluators. The scores for each variety were multiplied by the relative weight of a given trait to get the final result and then added with the results of other characters to find out the total score of a given variety.

### 2.4 Data analysis

The recorded agronomic data were subjected to the analysis of variance (ANOVA) using SAS, 9.2 version (SAS, 2009), and mean separation was carried out using the least significant difference (LSD) test at a 5% probability level. Before the combined ANOVA analysis, Bartlett's test for the homogeneity of the two-year error variances was examined. Farmers' preference data were analyzed using simple ranking methods per the given value (De Boef and Thijssen, 2007). The ranking was done on consensus where differences are resolved through discussion (De Boef and Thijssen, 2007).

## 3 Results and discussion

### 3.1 Evaluation of groundnut varieties for yield, yield components, and agronomic traits

Comparison of errors squares of the mean over two years showed homogeneity of variance. The analysis of variance (ANOVA) revealed highly significant to the significant varietal difference among the six groundnut varieties for all the traits studied (Table 2) indicating the presence of inherent variability among the varieties tested in the study area. Two varieties Bulki-01 and BaHajidu yielding other varieties tested in Taricha Zuriya district in the Dawuro zone (Table 2). The insignificance of interaction indicated that the yield of groundnut varieties did not differ over years in the trial district. Yield variations have been reported by different authors in legume crops. For instance, groundnut (Tulole et al., 2008; Biru and Daraje, 2014), common bean (Demelash et al., 2019).

**Table 2:** ANOVA on mean squares of phenology (flowering and maturity), yield, and yield components of groundnut varieties during the 2019 and 2020 main cropping seasons.

Source of variation	Df	SCE	SCH	Mean square of yield and yield components					Yield
				DF	DM	SPP	PPP	HSW	
Year	1	2.38	0.11	1.50	0.07	0.01	0.38	0.18	3025.14
Rep (year)	6	21.05	22.35	0.47	3.59	0.02	0.06	1.16	2941.37
Variety	5	21.52	22.60	36.51∗∗	666.10∗∗	0.75∗∗	29.19∗∗	230.39∗∗	242862.00∗∗∗
Year∗variety	5	5.46	10.84	1.12	6.42	0.03	1.45	9.33	1184.79
Error	78	14.27	17.02	0.71	7.59	0.02	0.21	1.69	4684.3
CV (%)	-	12.23	13.91	2.30	1.90	6.29	2.77	2.80	4.05
R^2^ (%)	-	19.15	18.5	77.80	85.10	70.97	90.42	90.16	77.25

Df = degree of freedom CV = coefficient of variation, R^2^ = coefficient of determination, ∗, ∗∗, ∗∗∗ = significant difference at 0.05, 0.01 and 0.001 respectively; SCE = stand count at 50% emergence, DF = days to 50% flowering, DM = days to 90% maturity, SPP = seeds per pod, PPP = pods per plant, HSW = hundred seed weight and SCH = stand count at harvest.

The mean of groundnut varieties in terms of stand count at 50% emergence, days to 50% flowering, days to 90% maturity, stand count at harvest, seeds per pod, pods per plant, hundred seed weight, and pod yield (kg ha^−1^) showed highly significant (P < 0.001) to significant differences (P < 0.05) and their values were illustrated in Tables 2 and 3. Varieties showed significant differences in the number of seeds per pod, the number of pods per plant, and the weight of 100 seeds. Relatively, the higher number of pods per plant was recorded for Sedi and BaHajidu varieties with 17.70, followed by Werer-963 with 16.80 pods per plant, respectively. In contrast, Werer-962 obtained a lower number of pods per plant (14.00). Groundnut varieties showed differences in the number of seeds per pod. The variety (Werer-963) had more seeds per pod (2.70), followed by Sedi (2.40) than the other varieties. There were considerable variations among the varieties in terms of 100 seed weight. BaHajidu had the highest 100 seed weight (50.20 g) while Sedi had the lowest 100 seed weight (40.30 g). This study agrees with other authors (Berhane et al., 2017; Fantaye et al., 2018) who reported variability among groundnut varieties depending on the season for yield components.

**Table 3:** Yield and yield-related traits performance of groundnut varieties at Taricha Zuria in 2019 and 2020.

Variety	Mean values of traits for each variety							
	SCE	DF	DM	SCH	SPP	PPP	HSW	Yield (kg ha^−1^)
Sedi	31.28ab	35.30c	133.70e	30.46a	2.40b	17.70a	40.34f	1605.30b
BaHajidu	30.61ab	38.83a	144.30b	30.20ab	2.20c	17.70a	50.20a	1805.84a
Manipinter	29.01b	36.50b	150.30a	27.33b	2.20c	16.50bc	47.80c	1588.50b
Bulki-01	31.58ab	36.80b	146.00b	30.24a	2.10c	16.44c	46.80d	1805.50a
Werer -962	*32*.26a	35.00c	135.80d	29.50ab	2.20c	14.00d	49.00b	1536.40c
Werer -963	30.06ab	34.84c	138.80c	30.23a	2.70a	16.80b	42.80e	1780.20a
Mean	30.80	36.20	141.50	29.66	2.30	16.5	46.2	1686.9
LSD (5%)	2.65	0.59	1.94	2.90	0.10	0.32	0.914	48.174
CV (%)	12.26	2.30	1.94	13.91	6.30	2.77	2.81	4.10

Means with the same letter (s) in the same column are not significantly different at P < 0.05; LSD = least significant difference; CV = coefficient of variation; SCE = stand count at 50% emergence, DF = days to 50% flowering, DM = days to 90% maturity, SCH = stand count at harvest.

Varieties Bulki-01 and BaHajidu were better yielded with their mean values of 1805.50 and 1805.84 kg ha^−1^, respectively (Table 3).

Relatively, the lower yield value was recorded for groundnut variety Werer-962. Varieties Manipinter and Sedi gave reasonable yield without significant yield differences among these varieties (Table 3). The highest seed yield (1805.84 kg ha^−1^) was recorded in BaHajidu, which yielded 17.53 % over Werer-962, which had the lowest (1536.30 kg ha^−1^) numerical yield and was statistically inferior to the other varieties. The yield differences of these varieties could be due to their differences in genetic characteristics and the nature of agroecological adaptability, which is in agreement with the conclusions of Bale et al. (2011) who pointed out that seed yield differences among varieties were attributed to higher efficiency in the manufacture and partitioning of assimilates to the reproductive sink, which in turn led to more grain yield formation.

Moreover, Alemayehu et al. (2014) reported that Sedi variety yielded a shelled seed of 2042–2944 kg ha^−1^ in eastern and southern Ethiopia, which is in contrast to the present findings. The significance of yield trait has been reported by many authors in different legume crops, such as groundnut (Amare and Tamado, 2014; Biru and Daraje, 2014; Aliyi, 2017; Wedajo and Wondewosen, 2017), common bean (Demelash et al., 2016; Teame et al., 2017), faba bean (Wondimu, 2016), chickpea (Yasin et al., 2017). A significant difference was observed between varieties in days to flowering and physiological maturity as presented in Table 3. The shortest number of days to 50% flowering (34.83) was observed in the variety Werer-963. Sedi matured in 133.60 days, followed by Werer-962 in 135.80 days, which were earlier than the other varieties.

The longest number of days to 50% flowering (38.80 days) and the third longer physiological maturity (144.30 days) were observed for the variety BaHajidu.

Earliness or lateness in the days to maturity might have been due to their inherited characters, early acclimatization to the growing area to enhance their growth and development. The observed difference in earliness traits (days to flowering and days to physiological maturity) were due to differences in genotype, environment, and the genotype by environment interaction as groundnut show variability in growth habit, seed characteristics, maturity, and adaptation.

This is in agreement with the report of Alemayehu et al. (2014), Berhane et al. (2017), and Mastewal et al. (2017) who reported variability among groundnut varieties across seasons for phenological traits in eastern and southern Ethiopia.

### 3.2 Farmer preferences in the selection of groundnut varieties

Table 4 shows the farmers' preferences and participants’ perceptions of the best and least preferred varieties for growing in their environments. The farmers agreed on their preferences for the best yielding varieties tested in groundnut experiments.

**Table 4:** Direct rank matrix by using traits preferred by farmers for selection of groundnut varieties in the 2019 and 2020 main cropping seasons.

Traits for selection	RW	Groundnut varieties and selection by farmers					
		Bulki-01	Werer-963	BaHajidu	Werer-962	Sedi	Manipinter
Pod yield	3	**12** (4)	**12** (4)	**15** (5)	**6** (2)	**9** (3)	**3** (1)
Seed color	2	**8** (4)	**8** (4)	**6** (3)	**2** (1)	**4** (2)	**4** (2)
Early maturity	3	**12** (4)	**6** (2)	**9** (3)	**3** (1)	**3** (1)	**15** (5)
Marketability	3	**12** (4)	**12** (4)	**9** (3)	**6** (2)	**6** (2)	**3** (1)
Adaptability	3	**12** (4)	**9** (3)	**12** (4)	**6** (2)	**6** (2)	**3** (1)
Resistance to insect pest	2	**6** (3)	**4** (2)	**8** (4)	**4** (2)	**6** (3)	**4** (2)
Resistance to disease	2	**8** (4)	**4** (2)	**8** (4)	**6** (3)	**4** (2)	**4** (2)
Pod number	2	**8** (4)	**6** (3)	**6** (3)	**2** (1)	**4** (2)	**4** (2)
Good taste	3	**12** (4)	**9** (3)	**9** (3)	**9** (3)	**6** (2)	**3** (1)
Pod size	2	**6** (3)	**6** (3)	**8** (4)	**4** (2)	**4** (2)	**2** (1)
Total score	26	96	76	90	48	52	45
Rank		1	3	2	5	4	6

Numbers in parenthesis are scores given by farmers to each variety for each character (5 = excellent 4 = very good, 3 = good, 2 = fair, 1 = poor); Numbers in the bold case are the product of the relative weight of the selection criterion and the score of a variety given by farmers. Ranks are in ascending order from one to six. RW = relative weight of traits given by farmers (1 = less important, 2 = important; 3 = very important).

They set the criteria; seed color, early maturity, marketability, adaptability, resistance to an insect pest, pod number, good taste, large pod size, high pod yielding, and resistant varieties to select the best variety.

Groundnut varieties preference using traits identified by the district farmers themselves showed that Bulki-01, BaHajidu, and Werer-963 were preferred by farmers. Bulki-01 (96) scored the highest value and Manipinter scored the lowest value (45). BaHajidu (90) and Werer-963 (76) were ranked by farmers as the second and third best varieties, respectively (Table 4). Likewise, comparing farmers’ results and researcher calculated yield data in Table 3, farmers' preferences for these above three varieties were superior in yield attributes. In addition, some participants selected early maturing varieties such as Sedi and Werer-962 for drought escape. In this study, farmers generally preferred high pod yielding, pest, and disease tolerant varieties. During discussions with the farmers, the color of the grain and the grain size characteristics were mentioned for marketability. Tolerance to biotic and abiotic stresses, earliness, marketability, cooking characteristics, seed color and size, and growth habit were important selection criteria (Berhane et al., 2017). Seltene et al. (2019) reported a similar preference criterion for groundnut production study in eastern Ethiopia. From the analysis of the data collected by the researchers, the performance of the same variety had better and the yield was higher. Farmers confirmed their ability to select well suitable and preferred varieties, in their circumstances, using their selection criteria.

## 4 Conclusion and recommendations

The results of the current study indicated that the groundnut variety BaHajidu had the highest mean pod yield, although not significantly different from yields obtained from varieties Bulki-01, and Werer-963. Yields from the mentioned varieties were statistically similar and were selected by farmers due to their better yield, seed color, marketability, and adaptability. Therefore, the varieties were selected for incorporating into the local farming systems.

The present selection process also verified that farmers were skillful in selecting important traits for grain yield and identity varieties best suited to their locality. Overall, participatory variety selection has been efficient and honest in identifying suitable crop varieties through partnerships with resource-poor farmers. These high-yielding and farmers’ preferred varieties Bulki-01, Werer-963, and BaHajidu were recommended for pre-extension demonstration and large-scale production for Taricha Zuriya district in the Dawro Zone and related agro ecologies.

## Declarations

### Author contribution statement

Demelash Bassa Belayneh: conceived and designed the experimets, performed the experiments, analyzed and interpreted the data, wrote the paper.

Yasin Goa Chondie: Designed and performed the experiments, edited the written paper.

### Funding statement

This research was supported by Agricultural Growth Program -II (AGP-II) project.

### Data availability statement

Data included in article/supplementary material/referenced in article.

### Declaration of interests statement

The authors declare no conflict of interest.

### Additional information

No additional information is available for this paper.
